# Validation of an Alternative Feather Sampling Method to Measure Corticosterone

**DOI:** 10.3390/ani10112054

**Published:** 2020-11-06

**Authors:** Marielu Voit, Roswitha Merle, Katrin Baumgartner, Lorenzo von Fersen, Lukas Reese, Mechthild Ladwig-Wiegard, Hermann Will, Oriol Tallo-Parra, Annaïs Carbajal, Manel Lopez-Bejar, Christa Thöne-Reineke

**Affiliations:** 1 Animal Behaviour and Laboratory Animal Science, Institute of Animal Welfare, Freie Universität Berlin, Königsweg 67, D-14163 Berlin, Germany; Lukas.Reese@fu-berlin.de (L.R.); Mechthild.Ladwig-Wiegard@fu-berlin.de (M.L.-W.); Christa.Thoene-Reineke@fu-berlin.de (C.T.-R.); 2 Institute for Veterinary Epidemiology and Biostatistics, Freie Universität Berlin, Königsweg 67, D-14163 Berlin, Germany; Roswitha.Merle@fu-berlin.de (R.M.); Hermann.Will@stadt.nuernberg.de (H.W.); 3 Tiergarten Nürnberg, Am Tiergarten 30, D-90480 Nuremberg, Germany; Katrin.Baumgartner@stadt.nuernberg.de (K.B.); lorenzo@vonfersen.org (L.v.F.); 4 Veterinary Faculty, Campus UAB, Universitat Autònoma de Barcelona, 08193 Bellaterra, Spain; Oriol.Tallo@uab.cat (O.T.-P.); anais.carbajal@uab.cat (A.C.); Manel.Lopez.Bejar@uab.cat (M.L.-B.); 5 College of Veterinary Medicine, Western University of Health Sciences, Pomona, CA 91766, USA

**Keywords:** feather corticosterone, comparative study, plucked feathers, cut feathers, Domestic Goose, Mulard Duck, animal welfare

## Abstract

**Simple Summary:**

Research projects on birds’ welfare or their stress physiology are often complemented by measurements of corticosterone level in feathers. Until now, the common standard for this method is to collect the feathers by plucking, a procedure which on living birds is presumed to be painful and to cause stress. Therefore, in most European countries an animal experiment application is required. The aim of this study was to validate an alternative, possibly less stressful sampling method: cutting the feathers close to the skin. The examined species were geese and ducks from a conventional poultry husbandry. There was no relevant difference between the two methods assessed according to statistical analysis. In conclusion, it is reasonable to assume that feather cutting could be established as an alternative sampling method for measuring corticosterone. Nevertheless, we recommend further research on other species to confirm these results.

**Abstract:**

The most common feather sampling method for feather corticosterone measurement is by plucking the feathers from the bird’s skin. This procedure performed on living, restrained birds is qualified as an animal experiment according to German/European legislation, which has to be applied for from the competent authorities. The Directive 2010/63/EU requires the full implementation of the 3-R Principle of Russel and Burch in animal experiments, which means not only to replace the use of animals, but also to reduce the number of animals used and to refine procedures whenever possible. In response to this issue, the aim of this study was to validate an alternative, less invasive sampling method by cutting feathers close to the skin in comparison to the gold standard of plucking them. For this proof-of-principle study, a conventional poultry husbandry with trial groups of geese (*Anser anser domesticus*) and ducks (*Anas sterilis*) was selected. All birds were kept under the same living conditions to standardize the influencing factors regarding husbandry, and thus, their stress levels. Feather samples were collected between the shoulders from 46 geese and 51 ducks, both by cutting as well as by plucking, directly after slaughter for meat production. Feather corticosterone levels were measured with Enzyme-Linked Immunosorbent Assay (ELISA). Results were compared using Bland–Altman plots and concordance correlation coefficients (CCC). It could be seen that concordance between corticosterone levels in cut and plucked feathers was rather poor: 0.38 for *Anser*, and 0.57 for *Anas*. However, comparing the mean corticosterone values in pg/mm of each species with their respective standard deviations, the differences between the methods were negligible. As the results showed that the differences between the individuals were markedly greater than the differences between the methods, the determination of corticosterone levels in cut feathers is valid compared to using plucked feathers. The validation tests of ELISA showed only acceptable repeatability and reliability. Hence, the results should be verified in further studies. In conclusion, it is recommended for future research to use cut instead of plucked feathers for corticosterone measurement.

## 1. Introduction

The technique of measuring corticosterone in feathers enables a long-term reconstruction of hormone exposure during feather growth. Feather growth has a timeframe ranging from days to weeks, as it depends on the species, the type of feather and the life history stage of the individual [1]. The steroid hormone corticosterone is released from the adrenal gland in baseline concentrations and in response to perceived or actual stress stimuli [1,2]. An acute rise in corticosterone concentration protects the bird by promoting those behaviors that reduce risk and is associated with a higher annual adult survival rate [3]. However, chronic high concentrations of corticosterone are detrimental to birds’ welfare and can lead to reproductive failure, low growth rate or neuron death [2,4,5,6]. In consequence, corticosterone serves as a very useful physiological indicator of the impact of stress stimuli for scientific investigations [7].

Plasma corticosterone is subject to diurnal variations and increases in stressful situations. Therefore, repeated blood samples are necessary to show an expressive hormone course. On one hand, it may result in underestimating hormone exposure because of hemodilution, already present after 15 min during the second bleeding [8]; on the other hand, it may cause a higher stress level due to repeated capture of the birds. Whereas measuring feather corticosterone is, especially in cut feathers, a non-invasive technique that gives the researcher the opportunity to gain a retrospective and long-term view of a bird’s corticosterone exposure. However, the method is limited in reflecting just the time of feather growth, whereby short-term stress cannot be estimated [1,9]. In conclusion, due to this fact, the measured values mirror only the sum of the hypothalamic-pituitary-adrenal axis (HPA axis) activity including the stress incidents that the bird experienced in this period.

Next to these two methods of corticosterone measurement, many reports exist about other matrixes that may be used as bases for stress-level studies. The examination of feces plays an essential role [10,11]. For correct interpretation of the corticosterone metabolites in birds’ droppings, the gut passage time needs to be known to draw conclusions about the timeframe of exposure. For example, Kotschral et al. describe a short gut passage time of two to three hours in domestic geese [12]. Eggs can also be used for hormone-level measurement to show stress in egg-laying birds during egg production [13,14]. Saliva has proved to be unreliable as an ideal medium for corticosterone measurements in birds [15]. In summary, it depends on the study objective which question is asked and thus, which medium is most suitable for the measurements.

Consequently, the amount of corticosterone embedded is correlated with the baseline level of corticosterone and stressor-related levels experienced by the bird during feather growth [1]. During molt, the feather follicles are supplied with blood to enable feather growth. As soon as the growth is accomplished the feather dries out, but the corticosterone is stored in the keratin. Therefore, the measured amount of corticosterone reflects the exposure to the hormone when blood circulates in the feather [1].

In recent studies, hormones have mainly been measured in plucked feathers. Using this sampling method presents a problem: the removal of feathers from living birds is traumatic and is thus interpreted as painful [16,17,18,19]. Gentle’s book ‘Pain in Birds’, lists feather removal as a painful intervention [18]. It describes behavioral and physiological changes to nociceptive stimulations: increasing heart rate and blood pressure, a high amplitude in the electroencephalogram and behavioral patterns related to pain [18]. Therefore, an animal experiment application is currently required in Germany, which partly implies a long delay between application and permission. However, most importantly, the welfare of the animal should be paramount. In consideration of Directive 2010/63/EU, the number of animal experiments should be reduced and the 3-R Principle should be followed [20,21]. In conclusion, the cutting of feathers would be an alternative and less-invasive method.

Not only do external stressors affect the hormone level but individual biological data are essential to interpret the corticosterone level. Romero and Fairhust elucidate that “corticosterone data is greatly improved if the researcher can characterize the basic ecophysiological state of the bird as close as possible to the period of hormone deposition” in the feather [1]. Therefore, it is important to include factors such as age, sex, body condition, activity, molt and other biometrics to understand and eventually standardize variation in feather corticosterone in the study [1]. Several projects describe different reactions of young birds to stressors resulting in varied increasing or decreasing blood corticosterone in comparison to adults [22,23]. In addition, sex may also be an influencing factor. Common Redpolls (*Acanthis flammea*) were tested and showed lower plasma levels in adult males than in females or young males [24]. Additionally, in Snow Petrels (*Pagodroma nivea*), stress-related plasma corticosterone correlated negatively with body condition in females, but not in males [25]. Thus, because corticosterone could be affected by body condition, the health of the animal should not be disregarded [26,27]. Furthermore, the external factors that should be mentioned as influencing the corticosterone level are the habitat and its quality, as well as environmental conditions, season, and feeding routine [9,28,29,30,31]. Hau et al. describe a variation of stress-induced corticosterone that is related not only to different body masses and survival rates, but also to lower levels commonly seen in species of colder and drier environments [3]. When conducting experiments, the trial group should be similar in its biological data and living conditions concerning aspects which induce possible corticosterone level changes. Thereby, the whole group experiences the same stressors and, thus, maintains the influencing factors equal according to their species-specific ethological needs. Hence, standardizing the influencing factors on corticosterone levels, as far as possible, and understanding the differences within the levels will be expected. Consequently, the comparison of two methods is carried out in a better way because the variation within and between the groups is kept as low as possible. Of course, individual differences in the baseline of corticosterone can still occur, as can specific stress-induced peaks.

Geese and ducks were selected for this study because the order Anseriformes is mentioned on different lists of bird species for which ethological and physiological research on the relevance of flying in assessing wellbeing is recommended [32,33]. Furthermore, conventional poultry farming was chosen because the birds are slaughtered for food purposes, there was a sufficiently large number of them and—most importantly—they were of the same age and experienced the same living conditions.

Nowadays, aspiring knowledge about different bird husbandries and their respective animal welfare concerns is a clear objective in zoological institutions. New research projects have been conducted to examine the physiological and behavioral effects of deflighting procedures on zoo birds to assess the birds’ wellbeing. One possibility is the measurement of corticosterone levels in feathers. The practice of deflighting birds in zoological institutions is regularly performed all around the world [34,35] but has become an increasingly pertinent issue due to legislative changes and increasing public awareness of animal welfare. Reese et al. give an overview of the relevance of this technique in combination with potential ethological and welfare concerns and furthermore pointing out the legal regulations in different countries [36]. As an example, in Germany, Article 6 of the Animal Welfare Act states that the total or partial amputation of parts of the body or the total or partial removal or destruction of organs or tissues of a vertebrate animal is prohibited [37]. In May 1998, the law was revised omitting the section on exceptions to the ban on amputation, “unless it is necessary for husbandries”. The practices of deflighting zoo birds are increasingly criticized all over Europe, even though each single country has a different legal interpretation. Many authors describe deflighting as a violation of animal welfare and the right to physical integrity should be legally protected [36,38,39,40,41]. However, from a different point of view, keeping birds deflighted could be, in some cases and in some species that are less dependent on or even independent of the ability of flight, considered necessary to protect them from escaping and to keep them in a better way according to their own biology [32,42]. The EU Zoos Directive 1999/22/EC defines the duties and responsibilities of zoological institutions as conservation, education and research. In conclusion, these institutions have, on the one hand, to take into account the individual needs of the respective species and, on the other, to prevent them from escaping [43]. Next to closed enclosures such as aviaries and ecosystem halls, deflighting birds for outdoor enclosures is often a way to enable birds to be kept in such institutions; therefore, a balance between the option to keep them in aviaries or in open-space enclosures and the welfare of the animals must be found [32,35]. Zoos are part of many European and international conservation breeding programs for which unified regulations concerning flightless birds and therefore science-based animal welfare assessments should be encouraged [32,36,43]. Associations such as the World Association of Zoos and Aquariums (WAZA) or, more regionally, the European Association of Zoos and Aquaria (EAZA), aim to introduce an international ethical code for zoos. Their guidelines provide a foundation for appropriate husbandries that should allow the animal to express its principal natural behaviors [44,45]. In addition, nationwide associations such as the German Veterinary Association for Animal Protection (Tierärztliche Vereinigung für Tierschutz—TVT) state the need for more ethological and physiological research into different husbandries [33].

In consequence, several ongoing research projects are evaluating different deflighting and husbandry practices of captive birds with respect to animal welfare concerns. In order to assess the wellbeing of these birds, the studies evaluate behavioral observations in combination with corticosterone measurements from feathers. This project aims to provide a basis for future studies on zoo birds and their relatives living in the wild.

The objective of this study was to investigate whether feather plucking could be replaced by feather cutting. In conclusion, after approval of the competent authority an animal experiment application is not necessary for cutting the feathers of living birds. Next to reducing the pain for the individual, another issue encourages the aim of this project. Following the protocol for the measurement of corticosterone in feathers, the calamus needs to be cut off as a first step of the protocol, even before measuring the total feather length of the sample. Regarding those parts of the feathers that are used for the analyses, they are in fact similar if either cut or plucked. Hence, the hypothesis is that there is no relevant difference in the corticosterone level between plucked and cut feathers and therefore cutting is a suitable alternative sampling method.

## 2. Materials and Methods

### 2.1. Subjects

For this project, two species of waterfowl of the order Anseriformes were chosen: The Domestic Goose (*Anser anser domesticus*) and the Mulard Duck (*Anas sterilis*)—a hybrid of the Muscovy Duck (*Cairina moschata domestica*) and the Pekin Duck (*Anas platyrhynchos domesticus*).

The feather sampling took place directly after slaughter. As a sampling site, the region between the shoulders was chosen. These geese and ducks had been kept in an open-air enclosure. Just catching them for the sampling would have been very stressful for the animals as would holding them tightly for feather removal. Despite this, the plucking of feathers itself is likely to be painful to the birds [17,18,19]. Removing the feathers directly after slaughter allowed for an organized allocation of the individuals to the two sampling methods and for plucking them without an animal experiment application, according to the 3-R Principle [20,21] and, as a positive effect, a shorter legal method without the processing time of an application.

To create a conclusive study for the comparison of plucked and cut feathers, there is the need to use a trial group with preferably similar characteristics [46,47,48]. In the chosen groups of ducks and geese, the individuals of each species were of the same age. Both chosen species did not show prominent sexual dimorphism, whereby this factor was not considered in this research project. However, sex disparity of corticosterone levels is often only put in context with different gender plumage hue [9,24,49,50,51]. Nevertheless, control of sex should be taken into account in further studies (if the sex is not obvious, e.g., via DNA analysis in feathers). The plumage of both species is mainly white in color.

### 2.2. Husbandry

According to the guidance of the “Standing Committee of the European Convention for the Protection of Animals Kept for Farming Purposes”, the husbandry of geese and ducks was evaluated as follows [47]:

All geese and duck chicks were bought from a wholesaler settled in northern Germany in mid-July 2018. The animals were transported in a conventional manner in accordance with transport legislation. At purchase, the age of the ducks was three weeks and the geese four to five weeks. They were transferred directly to a pasture measuring 5500 m² with an extension area of 4500 m², resulting in minimum 0.05 m²/individual of a total of 130 geese and 120 ducks. The pasture was enclosed with an electric fence. The ground consisted mainly of grassland; the rest was earthy. This gave the chicks the opportunity to search for plants and small invertebrates. The area was big enough to guarantee that the geese and ducks had enough space to flap their wings, to perform their eating and drinking habits and to allow a plumage plastering movement.

The enclosures were provided with several shelters, such as old tractor-trailers, with enough space for the whole group, which served as sun protection, as well as protection from other weather conditions, environmental hazards and birds of prey.

Several water troughs and many smaller water buckets were distributed in the pasture. The water in the containers was deep enough to cover the whole head with water, so every goose and duck had the opportunity to reach water to drink or to clean their plumage at all times. As an enrichment to the measure, there was the possibility to expand the ground to a small stream. The troughs were cleaned regularly once a day and moved around so that the ground did not get too muddy.

In the first months, the geese were separated from the ducks because of different feeding routines and composition between the two species (see Table 1). The geese were fed mainly with oats. In contrast, the ducks received wheat. In the first month, both received special breeding feed (Gallugold^®^ Enten-/Gänsekorn). Both always had free access to water and the pasture. They were held together in the enclosure in the last eight weeks before slaughter, resulting in a total fattening time of 4 to 5 months.

Prophylactic deworming was conducted twice in the time of rearing with levamisol (Concurat^®^-L 10%, Bayer Vital GmbH, Leverkusen, Germany; 40 mg levamisol per kg body weight dissolved in the drinking water).

There was a quarantine area available where sick individuals could be separated from the healthy flock. To assess the health of the whole poultry group, it was necessary to know the mortality rate (geese 2.3%, ducks 2.6%). In conclusion, the poultry keeper checked on the geese and ducks at a minimum twice a day to evaluate their health and body condition and to ensure the supply of water and food. Through the regular controls, the animals became quite accustomed to contact with humans.

To calculate the stage of feather growth for this research project, it was important that the molting of cover feathers was observed around six weeks before slaughter and at an estimated age of 4 to 5 months. The slaughter and feather sampling were performed at least six weeks after molting in order to guarantee sufficient feather growth. Slaughter took place on 18 November and December 2018. Catching before transportation took place directly before the slaughter date. Food and water were withheld only during transportation. Special poultry transport boxes were used for the journey. Two geese or four ducks were transported in each box. The distance from the enclosure to the slaughterhouse was around 9 km. During the slaughtering process, the birds were first placed in funnels and then stunned by a blow to the head. Afterwards, they were bled through a throat cut.

### 2.3. Sampling Protocol

Feather sampling took place directly after slaughter. The initially sampled 46 geese and 51 ducks were selected randomly from the total of 130 geese and 120 ducks. Three to five feathers with a minimum collective length of 20 cm per sample were both plucked and cut from every single individual between the shoulders. Three or four feathers from each goose and four to five feathers from each duck were collected for each sampling method: plucked and cut. While plucking, the researcher had to be careful that the feathers did not contain any blood and that the quills were dry. The cover feathers needed to be clipped as close as possible to the skin. In addition, to standardize the sampling site between the birds, every feather sample was cut and plucked from the interscapular region.

To protect the feathers from contamination the person in charge of sampling was wearing gloves. The researcher also tried to ensure that no other biological fouling, such as feces or blood, was present. The feather samples were stored in paper envelopes at room temperature [1].

### 2.4. Analysis of the Corticosterone Level in Feathers

For measuring the corticosterone in feathers, the protocol of Bortolotti et al. (2008) modified by Monclùs et al. (2017) was followed [9,52]. Both studies follow the recommendations of Buchanan and Goldsmith (2004) because each assay technique needs to be fully validated for each new species to get comprehensible results [53].

To synchronize the results, a minimum length of 200 mm per sample was required. Three to five feathers were selected of the same type, thus morphologically identical, and from every individual. Initially, following the protocol, the calamus of the plucked feathers was cut off [9,52,53].

A ball mill (Retsch^®^, MM200 type with two balls and 25 Hz) was used to mill the feather samples to a particle size of <2 mm. The duration of the process depended on the feather length: small feathers four minutes, larger ones 5 min. Due to the relatively different feather length between individuals, the weight of powder was recorded to the nearest 0.1 mg. A quantity of 1.5 mL of methanol was added to the feather powder of each sample and then mixed in a vortex (Vortex Mixer S0200–230 V-EU; Labnet International, Edison, NJ, USA) at room temperature for 30 min. The subsequent incubation was at 37 °C for 18 h (G24 Environmental Incubation Shaker, New Brunswick Scientific, Edison, NJ, USA). Afterwards, the mixture was centrifuged at 3500× *g* for 15 min (Hermle Z300K; Hermle^®^ Labortechnik, Wehingen, Germany). One milliliter of the supernatant was pipetted into an Eppendorf^®^ tube and dried in an oven (Heraeus Function Line T6^®^, Thermo Fisher Scientific, Waltham, MA, USA) at 38 °C until all liquid had evaporated. After the drying process, the residue was mixed with 0.25 mL of the buffer solution (containing BSA, NaCl, EDTA and Azide) delivered with the commercial enzyme immunoassay kit (ELISA Neogen^®^ Corporation, Ayr, UK). The composite was shaken in the vortex for one minute and then frozen at −20 °C until analysis. The corticosterone was measured as described by the manufacturer.

### 2.5. Statistical Analysis

First, the accuracy of the ELISA was proven by creating an intra- and inter-assay coefficient of variation (CV), the linearity of dilution and the measure of fit, R². The CV was created by pooling ten different samples from ten different individuals. The pool-CV was run three times per assay. Whereas the actual samples had only run the assay once.

An initial sample size of at least 45 animals in each group was chosen to conduct equivalence testing. An equivalence test of means using 45 sampling pairs achieves 90% power at the 5.0% significance level when the true difference between the means is 0.0, the standard deviation of the paired differences is 6.0, and the equivalence limits are −3.0 and +3.0.

The feather samples of this study had a large variance in size (in mm) as well as in mass (in mg). There are studies that use the size while other studies use mass. The feather growth rate is considered to be rather uniform between and within species [54]. Consequently, the hormone exposure is time-dependent and feather corticosterone should be standardized by length [1,9,26,55]. Thus, the chosen unit of this study was pg corticosterone/mm feather.

Two analyses were carried out in order to assess agreement between plucked and cut feathers: the concordance correlation coefficient (CCC) and the Bland–Altman plot. Consequently, the corticosterone values of each method were compared within the same individual.

The CCC is a measurement of precision and accuracy [56]. It ranges from 0 to 1 and reflects the degree to which the two observed values correspond to the 45° line through the origin that indicates perfect agreement [57].

**Figure 1:**
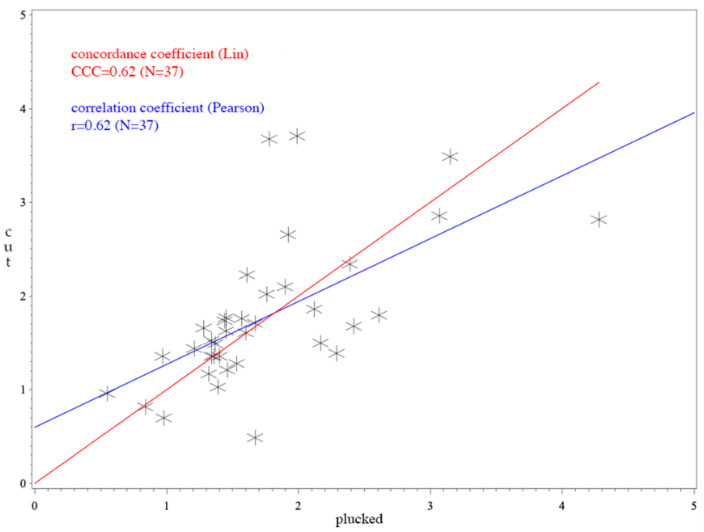
Concordance correlation coefficient (CCC) and Pearson’s correlation coefficient of *Anas*; *p*-value for Pearson’s correlation coefficient: *p* = 0.6111 × 10^−6^; the x-axis shows the corticosterone values of plucked feathers. The y-axis represents the values of cut feathers. The red line illustrates the concordance coefficient. The correlation coefficient is expressed in the blue line.

**Figure 2:**
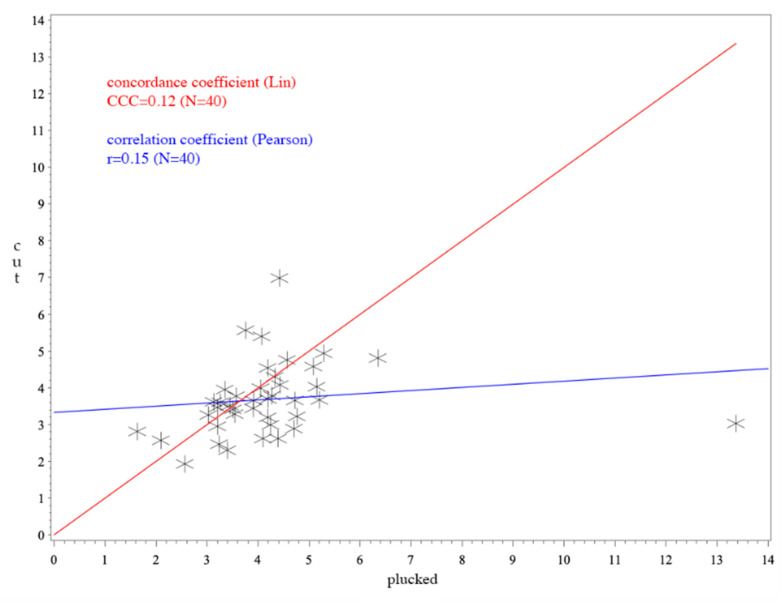
CCC and Pearson’s correlation coefficient of *Anser*; *p*-value for Pearson’s correlation coefficient: *p* = 0.4297; the outlier of 13.37 pg/mm is included. The x-axis shows the corticosterone values of plucked feathers. The y-axis represents the values of cut feathers. The red line illustrates the concordance coefficient. The correlation coefficient is expressed in the blue line.

**Figure 3:**
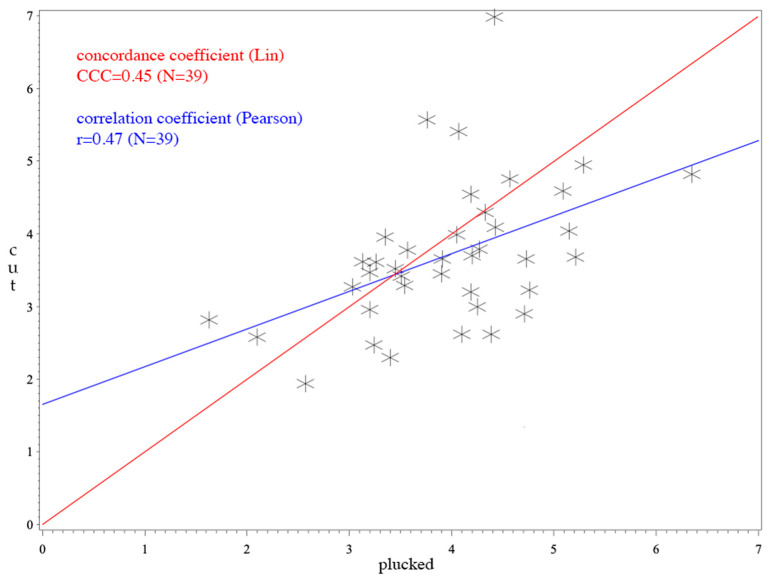
CCC and Pearson’s correlation coefficient of *Anser*; *p*-value for Pearson’s correlation coefficient: *p* = 0.0023; the outlier was excluded which leads to a total number of 39 individuals. The x-axis shows the corticosterone values of plucked feathers. The y-axis represents the values of cut feathers. The red line illustrates the concordance coefficient. The correlation coefficient is expressed in the blue line.

The Pearson’s correlation coefficient is also included in the analysis (see Figure 1, Figure 2 and Figure 3). This value describes a linear relationship between two variables. When its value is 0, there is no linear relationship and the line in the scatterplot is horizontal. However, the value does not describe a cause-and-effect principle and therefore was not considered helpful in this study.

**Figure 4:**
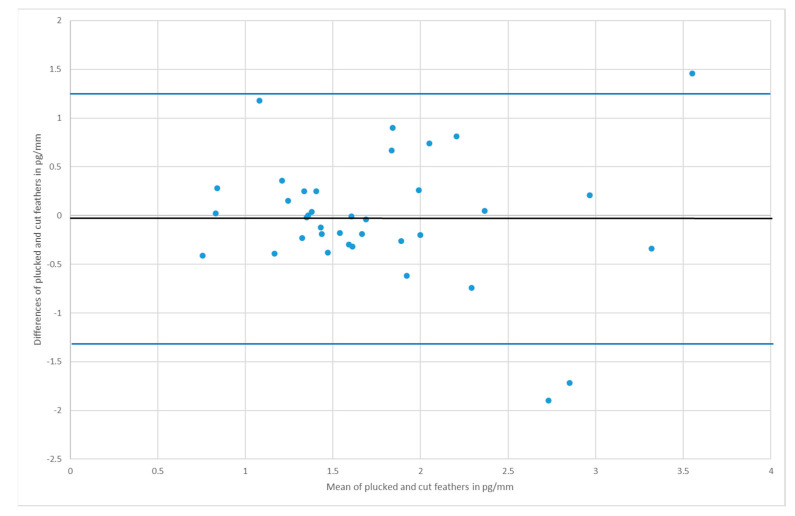
Bland–Altman plot of *Anas*; the residual plot displays the differences (y-axis) against the means of values (x-axis). The blue horizontal lines represent the mean difference ± 2 SD (mean = −0.03, SD = 0.65).

**Figure 5:**
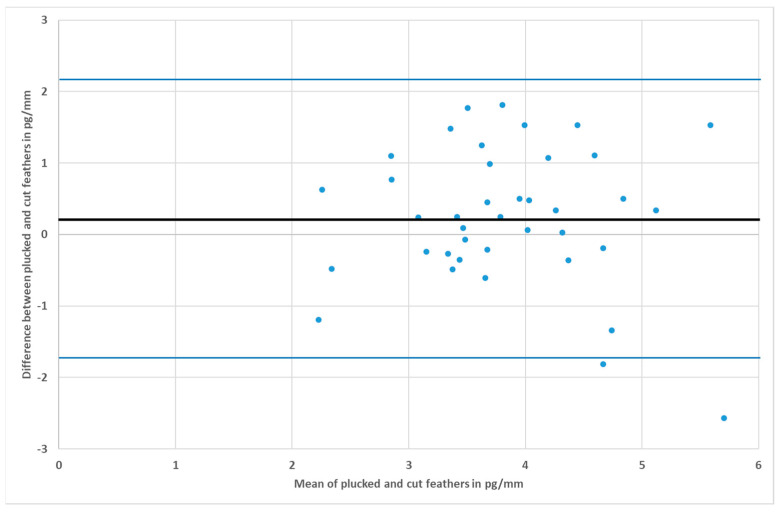
Bland–Altman plot of *Anser* without the outlier; the residual plot displays the differences (y-axis) against the means of values (x-axis). The blue horizontal lines represent the mean difference ± 2 SD (mean = 0.25, SD = 0.97).

The Bland–Altman plot, also known as the “difference plot”, displays the agreement of two measurement techniques [58,59]. The differences between the methods per sampling pair are plotted in a scatter diagram against the respective means of values (see Figure 4 and Figure 5). The middle horizontal line corresponds to the mean difference. The upper and the lower lines are the 95% confidence interval. In summary, Bland–Altman is a graphical method to assess the relationship between differences and to identify outliers. If the hypothesis is confirmed, the points will lie within the interval and scatter evenly around the centerline. Furthermore, there should be no ascending or descending tendency in the image.

## 3. Results

In *Anser*, the intra-assay CV amounted to 8.29% and the inter-assay CV to 15.79%. For *Anas*, a value of 8.34% for intra-assay CV and 14.25% for inter-assay CV was calculated. The linearity of dilution was determined by using 1:1, 1:2, 1:5 and 1:10 dilutions of pools with EIA buffer and by calculating the measure of fit R². The R²-value of *Anser* was 99.72% and that of *Anas* was 99.44%. The values of the spike-and-recovery test amounted to 111.08% ± 8.59% (*Anser*) and 116.40% ± 12.39% (*Anas*).

Due to the sampling conditions during the slaughter process, it was unavoidable that some samples had traces of blood and feces above the feathers; also, a few feathers had blood inside the calamus. This occurred in samples of 20 individuals, six of *Anser* and 14 of *Anas*. The contaminated individuals were excluded from the subsequent analyses [58,59]. This led to a total number of only 40 from *Anser* (initial number of 46) and 37 from *Anas* (initial number of 51).

The description of the feather characteristics and corticosterone levels in a comparison of the two methods is displayed in Table 2.

Since the probability of belonging to the distribution of values was only 5.49 × 10^−24^, one corticosterone value of *Anser* (13.37 pg/mm) was regarded as an outlier and was excluded.

For *Anas*, the CCC was 0.62 (see Figure 1) and for *Anser* it was 0.45 (see Figure 3). The average difference between cut and plucked feathers for *Anas* were −0.0251 with a standard deviation of 0.65 (see Figure 4). This resulted in a 95% confidence interval ranging from −1.2899 to +1.2396. Concerning *Anser*, the mean difference was 0.2544 with a standard deviation of 0.97 and a 95% confidence interval ranging from −1.6529 to +2.1617 (see Figure 5). The total average value of corticosterone of every single sample, whether cut or plucked, was at 1.76 pg/mm for *Anas* and at 3.83 pg/mm for *Anser*.

## 4. Discussion

The main result of this study shows that no relevant difference in corticosterone was found between feather plucking, the commonly established standard so far, and the alternative method examined of feather cutting. Hereafter, remarkable results of the statistical analysis are pointed out from which conclusions can be drawn.

A standardized sampling region was chosen even though the paper “Carotenoid-based plumage coloration reflects feather corticosterone levels in male House Finches (*Haemorhous mexicanus*)” by Lendvai et al. (2013) showed no difference in hormone levels between tail and breast feathers. However, there are studies on the various levels of absolute hair cortisol in different body regions in chimpanzees (*Pan troglodytes*) and Canada Lynx (*Lynx canadensis*), in which the researchers suggest standardizing the sampling region [60,61]. In addition, it was ensured that only white feathers were analyzed, because Tallo-Parra et al. (2015) describe choosing hair samples homogenous in color and body region in dairy cows [62].

Regarding the sample size, by excluding the contaminated animals, fewer animals are in the statistical evaluation compared to the number (minimum 45) which was calculated at the beginning. Therefore, the desired accuracy could not be maintained and the results should be interpreted with caution. In the future, more animals should be planned as a reserve. Considering the selection, the sampling strategy was indeed rather convenient, but there is no obvious reason for selection bias.

Firstly, the precision of ELISA was assessed by calculating the intra-assay CV from all duplicated samples and the inter-assay CV from samples running different ELISA tests. The specificity was evaluated with the linearity of dilution and the measure of fit, R². Furthermore, the spike-and-recovery test was applied to assess accuracy, which was calculated by adding a known amount of analyte to different volumes of pure standard cortisol solution. Only one value did not correspond to the required results: the inter-assay CV of *Anas* was just above the 15% reference point, from which a non-optimal inter-assay performance can be concluded [63,64,65]. However, in summary, the validation tests confirmed that it is possible to detect corticosterone concentrations in feathers of *Anser* and *Anas* with an acceptable repeatability and reliability by using ELISA.

With the chosen unit pg/mm, the corticosterone values were not as widely distributed as those expressed in pg/mg (Table 2). Due to this lower dispersion, we considered the values expressed in pg/mm to be more precise, considering the time-dependent aspect, and more appropriate than those in pg/mg [9].

Comparing the two different sampling methods, plucking resulted in values 0.27 pg/mm higher in *Anser* (7.1% of the total average value), whereas in *Anas* the cut feathers showed values only 0.02 pg/mm higher (0.6% of the total average value). In consequence, a statement that plucked feathers produce generally higher or lower values could not be supported. With a comparison of the mean difference between methods of 0.03 pg/mm (*Anas*) vs. 0.25 pg/mm (*Anser*) and the respective standard variations of 0.65 (*Anas*) and 0.97 (*Anser*), it becomes clear that there is no systematic difference between the methods that would lead to a systematic under- or overestimation. Hence, no general conclusions can be drawn about the effects on the corticosterone levels of one method or the other, and the differences between the methods can be said to be negligible.

A value of CCC under 0.9 is regarded poor, 0.9–0.95 moderate, 0.95–0.99 substantial and above 0.99 almost perfect [56]. Thus, the CCCs observed have to be regarded as poor in total. The CCC of *Anser* (Figure 3) was not as high as that of *Anas* (Figure 1). The higher value of CCC (0.62) implies a better agreement in *Anas*. This cocanuld also be observed in the Bland–Altman plot. However, it must be considered that CCC mainly assesses methodological differences using an identical sample. This is not the case in our study because the feathers were indeed from the same individual, but they were biologically different between the two sampling methods. Nevertheless, in terms of comparison with the mean values, the differences can be considered marginal.

In total, *Anser* produced a higher amount of corticosterone (3.82 pg/mm), whereas *Anas* had an average of 1.76 pg/mm. This was also reflected in the mean differences of the single values: 0.25 pg/mm for *Anser* and −0.03 for *Anas*. Additionally, *Anas* had a substantially lower standard deviation. Due to the larger differences of *Anser* and the more similar values of *Anas*, there is an overall better match of this study regarding *Anas*.

However, looking at the plot of *Anas* (Figure 4), three values were beyond the limits. Nevertheless, the measured results scattered more or less perfectly around the mean. Although the agreement in *Anser* was not as good as that in *Anas*, the Bland–Altman plot showed a fairly good agreement (Figure 5).

In conclusion, the agreement between plucked and cut feathers was rather poor, mainly concerning the CCC. However, taking into consideration the large variation in corticosterone levels within species, especially in the group of geese, and that there was no greater difference between the methods regarding all subjects, or no general trend of one group having higher levels than the other, it can be assumed that the poor agreement is rather due to general data variation. In addition, in contrast to the CCC, the agreement in the plot can be interpreted as more appropriate. Thus, cut feathers seem to be as suitable as plucked feathers.

Nevertheless, it must be added that the large variation of values within a species, as mentioned above, may also be explained with the following drawbacks of this study. Although the sampled feathers were standardized as far as possible (interscapular region, same color, same morphology, similar total length), the standardization is not as optimal as comparing a precisely identified feather between the subjects, for example the first wing feather. However, since the study should also serve as a basis for projects with wild birds, the sampling of a flight or tail feathers would be considered very critical because of their influence on the ability of flight. Therefore, we have chosen the sampling site between the shoulders. However, in the future, several cut feather samples from one individual should have to be analyzed in order to better assess the individual variation of the corticosterone levels. The same applies to the plucking method. Only then could the variation within a method be compared with the variation between methods and thus, draw conclusions about the possible negligibility of the discrepancy between methods. Feather corticosterone has been validated for tracking stress in several studies [1,9,26,66]. The purpose of this study was to test agreement between cut and plucked feathers within the same animals. The animals used were assumed to be not chronically stressed. To assess the relevance of the within-individual variation (median 15.6% for *Anser*, 17.8% for *Anas*), this and the maximum values (6.99 pg/mm in *Anser*, 4.28 pg/mm in *Anas*) must be compared to values from animals kept under different husbandry conditions. Since there are no values from stressed *Anser* and stressed *Anas* available in the literature, we discuss results of a study performed on Greater Flamingos for an estimation [48]. Stress was expected in one group due to repeated attacks by cranes. This group had a mean corticosterone level of 15.96 pg/mm and a maximum value of 20.93 pg/mm. The group with the smallest corticosterone values had a mean of 5.62 pg/mm. Although the absolute values cannot be compared between species, it seems that the differences between stressed and unstressed birds are much greater than the observed differences of 15–18%. This leads to the conclusion that although the statistical agreement between cut and plucked feathers was not satisfactory, the variation is not biologically relevant because all values were in a range below those expected in stressed animals. In addition, regarding this study, through the large standard deviation in corticosterone itself, the value alone allows us no general statement about the individuals’ stress. Therefore, corticosterone values should always be interpreted in combination with additional data, for example behavioral observations, or after repeated measurements.

## 5. Conclusions

The less-invasive method of cutting feathers seems to work as an alternative to plucked feather samples. Nevertheless, we refer to ‘less-invasive’, because it should not be forgotten that the birds must also be caught and restrained while cutting the feathers. This method should therefore only be used, if possible, if the bird is caught for other purposes anyway. However, the concordance, especially regarding the CCC values, was rather poor. Therefore, it is very important to encourage further studies to underpin the significance of this project. In future projects we would like to cut feathers from live birds instead of plucking them and thus make a contribution to the animal in the sense of refinement. Additionally, we hope that further studies can confirm this result in other bird species. Furthermore, other factors influencing the corticosterone level should be evaluated; for example, gender, as this factor was not taken into account in this study. Once a larger set of data has been established and cutting feathers is a suitable alternative, the influence of different husbandry conditions should play a major role in subsequent projects. Concerning this topic, ongoing studies, as well as currently published studies, focus on the evaluation of deflighting zoo birds, the comparison between different husbandries and the welfare implications. These deal with the species Greater Flamingo (*Phoenicopterus roseus*) [48] and Great White Pelican (*Pelecanus onocrotalus*) (ongoing study). The planning, structure and realization of research projects compared to these could also be expanded and simplified with this non-invasive sampling method.

Further studies to confirm the results of this study are planned on wild species as well as young birds.

## Figures and Tables

**Table 1:** Feeding schedule: overview of the different phases of the rearing feeding of the two species Domestic Goose (*Anser anser domesticus*) and Mulard Duck (*Anas sterilis*).

Fattening Period	Geese	Ducks	Additional Information
1. About two weeks	Crushed oats, pressed feed (Gallugold^®^ Enten-/Gänsekorn), fattening feed for geese (Gallugold^®^ Enten-/Gänsekorn)	Crushed wheat, pressed feed (Gallugold^®^ Enten-/Gänsekorn)	Periods 1 to 4: Water and pasture ad libitum
2. About two weeks	Oats, pressed feed (Gallugold^®^ Enten-/Gänsekorn)	Wheat, pressed feed (Gallugold^®^ Enten-/Gänsekorn)	Periods 1 to 3: Geese and ducks separated
3. One to two months	Oats	Wheat	
4. Final weeks before slaughter	Oats, wheat	Oats, wheat	Period 4: Geese and ducks together

**Table 2:** Overview of the measured results of 40 geese and 37 ducks in length, weight and corticosterone levels; the contaminated samples were excluded. The values in brackets are those including the outlier.

	Length in mm				Weight in mg				Corticosterone in pg/mg				Corticosterone in pg/mm			
	*Anser*		*Anas*		*Anser*		*Anas*		*Anser*		*Anas*		*Anser*		*Anas*	
	Plucked	Cut	Plucked	Cut	Plucked	Cut	Plucked	Cut	Plucked	Cut	Plucked	Cut	Plucked	Cut	Plucked	Cut
Mean	222	217	224	225	110.3	106.0	82.0	78.7	8.01 (8.42)	7.59	4.78	5.16	3.96 (4.20)	3.69	1.75	1.77
Max	249	242	251	248	135.2	134.0	109.0	106.0	12.90 (24.49)	11.54	10.88	12.24	6.35 (13.37)	6.99	4.28	3.71
Min	202	201	201	202	74.0	56.6	53.8	52.4	3.85	4.73	1.76	1.38	1.63	1.94	0.55	0.49
